# Gambogic acid induces apoptosis in diffuse large B-cell lymphoma cells *via* inducing proteasome inhibition

**DOI:** 10.1038/srep09694

**Published:** 2015-04-08

**Authors:** Xianping Shi, Xiaoying Lan, Xin Chen, Chong Zhao, Xiaofen Li, Shouting Liu, Hongbiao Huang, Ningning Liu, Dan Zang, Yuning Liao, Peiquan Zhang, Xuejun Wang, Jinbao Liu

**Affiliations:** 1 State Key Lab of Respiratory Disease, Protein Modification and Degradation Lab, Departments of Pathophysiology and Biochemistry, Guangzhou Medical University, Guangdong 510182, China; 2 Guangzhou Research Institute of Cardiovascular Disease, the Second Affiliated Hospital, Guangzhou Medical University, Guangzhou, Guangdong 510260, People's Republic of China; 3 Division of Basic Biomedical Sciences, Sanford School of Medicine of the University of South Dakota, Vermillion, South Dakota 57069, USA

## Abstract

Resistance to chemotherapy is a great challenge to improving the survival of patients with diffuse large B-cell lymphoma (DLBCL), especially those with activated B-cell-like DLBCL (ABC-DLBCL). Therefore it is urgent to search for novel agents for the treatment of DLBCL. Gambogic acid (GA), a small molecule derived from Chinese herb gamboges, has been approved for Phase II clinical trial for cancer therapy by Chinese FDA. In the present study, we investigated the effect of GA on cell survival and apoptosis in DLBCL cells including both GCB- and ABC-DLBCL cells. We found that GA induced growth inhibition and apoptosis of both GCB- and ABC-DLBCL cells *in vitro* and *in vivo*, which is associated with proteasome malfunction. These findings provide significant pre-clinical evidence for potential usage of GA in DLBCL therapy particularly in ABC-DLBCL treatment.

Diffuse large B-cell lymphoma (DLBCL), an aggressive form of non-Hodgkin's lymphoma (NHL), accounts for approximately 30%–40% of all NHL^1^. There are three subcategories in DLBCL: activated B-cell-like DLBCL (ABC-DLBCL), germinal center B-cell-like DLBCL (GCB-DLBCL) and primary mediastinal DLBCL (PMBCL)^2^,^3^. These subtypes are characterized by distinct differences in survival, chemoresponsiveness, as well as dependence on signaling pathways, especially the nuclear factor-κB (NF-κB) pathway. In particular, the ABC-DLBCL subtype, which is NF-κB-dependent, appears to have the worst prognosis among the three subtypes^4^,^5^,^6^. Patients with the ABC-DLBCL tend to have the poorest 5-year survival rate (16%), compared to GCB-DLBCL (76%) and PMBCL (64%)^7^. Treatment for DLBCL has been improved over the last decade, especially with the development of Rituximab, an anti-CD20 monoclonal antibody, in combination with CHOP (Cytoxan, Hydroxyrubicin, Oncovin, and Prednisone) therapy program^8^,^9^. Unfortunately, adverse events including bronchospasm, hypotension, cardiac arrhythmias and renal failure occur during the therapy. Furthermore, at least 25–30% of patients experience disease recurrence and patients with the ABC-DLBCL subtype is much more resistant to current treatment regimens^10^,^11^. Resistance to the Rituximab-CHOP (R-CHOP) therapy program develops over time and is becoming an emerging problem for DLBCL treatment. Therefore, the development of innovative therapies and identification of more effective drugs for DLBCL are clearly needed.

Gambogic acid (GA), a small molecule extracted from the traditional Chinese medicine gamboges^12^, has been approved by Chinese FDA for phase II clinical trial in solid tumor therapy^13^,^14^. Unlike other chemotherapeutics, GA has very low toxicity to the hematopoietic system^15^,^16^. Several molecular targets of GA have been proposed^17^,^18^. Most recently, we have reported that GA is a novel tissue-specific proteasome inhibitor, with potency comparable to bortezomib but much less toxicity^19^. Although proteasome inhibitors such as carfilzomib have been reported to induce cell death in DLBCL cells combining with HDAC (histone deacetylase) inhibitors^20^, the effect of GA on DLBCL remains unknown.

Here, we investigated the effects of GA in DLBCL cell lines and in mouse models. Strikingly, GA displays pronounced antineoplastic activity in both GCB- and ABC-DLBCL cells and in *in vivo* DLBCL xenograft models.

## Results

### GA inhibits cell proliferation in both GCB-DLBCL and ABC-DLBCL cells

**Figure 1:**
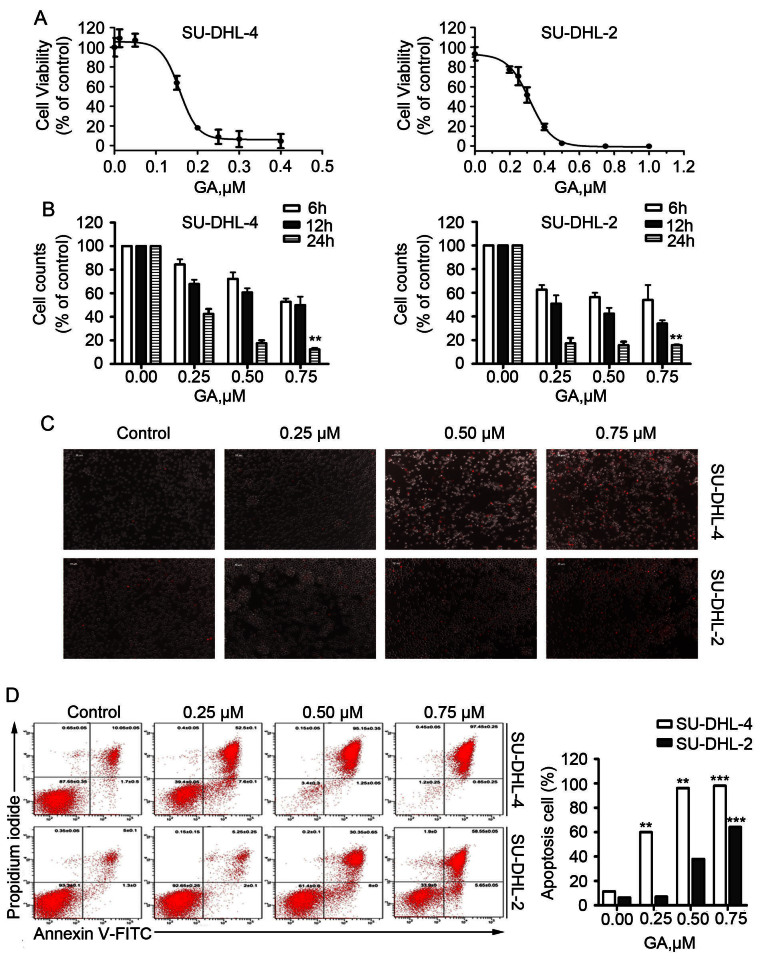
GA induces apoptosis in both GCB- and ABC-DLBCL cells. (A) GA decreases cell viability of SU-DHL-4 and SU-DHL-2 cells. SU-DHL-4 and SU-DHL-2 cells exposed to GA in various concentrations for 48 hours were subjected to MTS assay. Graphs represent data from three repeats. Mean ± SD (n = 3). (B) GA treatment inhibits cell proliferation in both GCB- and ABC-DLBCL cells. SU-DHL-4 and SU-DHL-2 cells grown in 24-well plates were treated with GA in various concentrations for 6, 12 or 24 hours. Total cell number was detected by trypan blue exclusion staining. Mean ± SD (n = 3). (C) GA induces cell death in GCB- and ABC-DLBCL cells. SU-DHL-4 and SU-DHL-2 cells were treated with different doses of GA for 24 hours, then propidium iodide (PI) was added to the culture medium and the PI-positive cells were recorded under an inverted fluorescence microscope. Representative images were shown. (D) GA induces apoptosis in GCB- and ABC-DLBCL cells. SU-DHL-4 and SU-DHL-2 cells were treated with GA at the indicated doses for 24 hours and apoptosis was detected using Annexin V-FITC/PI double staining with flow cytometry. Representative images (left) and pooled data (right, mean ± SD, n = 3) were shown.

SU-DHL-4 (GCB-DLBCL) cells are sensitive, while SU-DHL-2 (ABC-DLBCL) cells are very resistant, to R-CHOP therapy^3^,^21^. To investigate the effect of GA on the growth of DLBCL cells, SU-DHL-4 and SU-DHL-2 cells were treated with GA *in vitro* for 48 hours and cell viability was detected by MTS assay. As shown in Figure 1A, GA dose-dependently decreased the cell viability in SU-DHL-4 and SU-DHL-2 cells with IC_50_ values of 0.16 μM and 0.30 μM, respectively.

We next analyzed the kinetics of the capacity of GA to inhibit cell growth in GCB- and ABC-DLBCL cell lines. SU-DHL-4 and SU-DHL-2 cells were exposed to GA followed by trypan blue exclusion staining, a time- and dose-dependent decreasing proportion of total cells was observed by recording the total number of both trypan blue-positive and -negative cells (Figure 1B).

### GA induces cell death in both GCB- and ABC-DLBCL cell lines

We then examined the ability of GA to induce cell death in GCB- and ABC-DLBCL cell lines. SU-DHL-4 and SU-DHL-2 cells were treated with escalating concentrations of GA, followed by recording the PI-positive cells with fluorescence microscopy (Figure 1C) or by Annexin V/PI staining coupled with flow cytometry (Figure 1D). A dose-dependent cell death was observed.

### GA induces caspase activation in both GCB- and ABC-DLBCL cells

**Figure 2:**
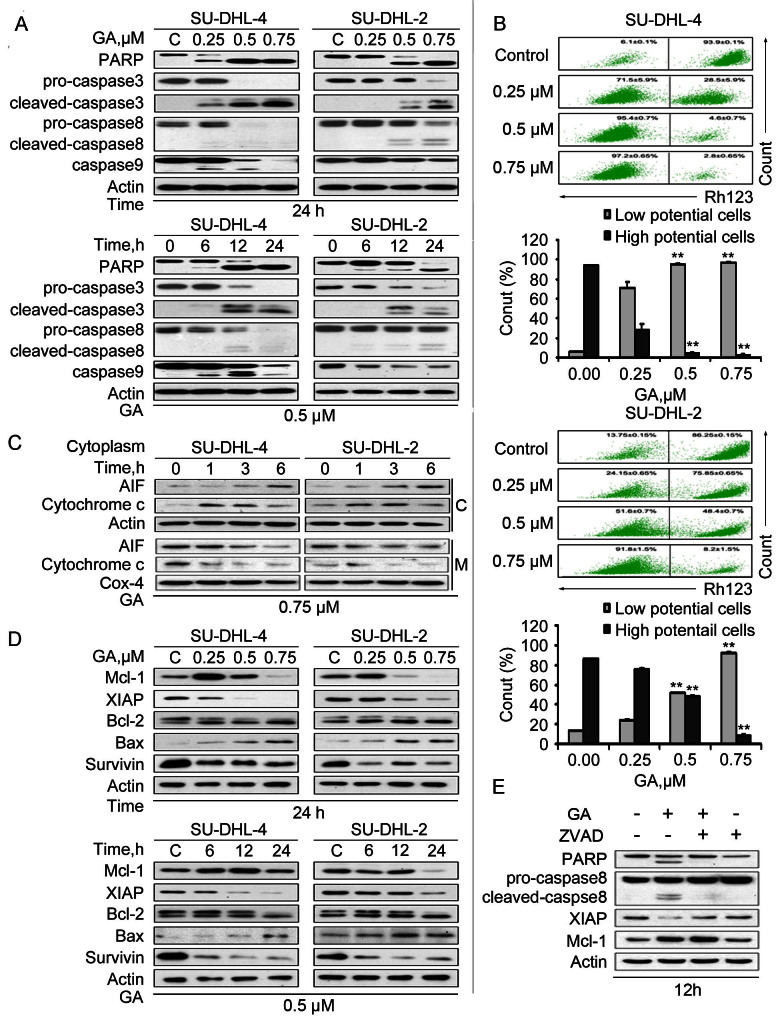
GA-induced apoptosis is associated with caspase activation and decreased expression of anti-apoptotic proteins in both GCB- and ABC-DLBCL cells. (A) GA induces cleavage of PARP and caspase−3, −8, −9 in SU-DHL-4 and SU-DHL-2 cells. Cells were treated with GA at the indicated dose for the indicated time, PARP and caspase−3, −8, −9 cleavage were analyzed with western blots. Actin was used as a loading control. C: control. (B) GA induces down-regulation of mitochondrial membrane potential in SU-DHL-4 and SU-DHL-2 cells. Cells were treated with 0.25, 0.5 and 0.75 μM GA for 24 hours, mitochondrial membrane potential were detected using rhodamine-123 staining coupled with flow cytometry, mean ± SD (n = 3). (C) GA induces AIF and cytochrome C release. SU-DHL-4 and SU-DHL-2 cells were exposed to GA for 1, 3 or 6 hours; then the cytosolic and mitochondrial fraction were extracted by digitonin buffer and Mitochondria Isolation Kit, respectively, and AIF and cytochrome C were detected with western blot analyses. Cox-4 was used as a loading control for the mitochondrial fraction. (C: cytosolic fraction; M: mitochondrial fraction.) (D) GA decreases the expression of anti-apoptotic proteins in SU-DHL-4 and SU-DHL-2 cells. Cells were dose- and time-dependently treated with GA. The anti-apoptotic proteins Mcl-1, XIAP, Bcl-2, survivin and pro-apoptotic protein Bax were analyzed by western blot. (E) GA decreases XIAP in a caspase-dependent manner. SU-DHL-2 cells were treated with 0.5 μM GA with or without caspase inhibitor z-VAD-fmk (20 μM) for 12 hours. The Mcl-1 and XIAP proteins were detected using western blot analyses.

SU-DHL-4 and SU-DHL-2 cells were then exposed to GA, followed by measurement of apoptosis-associated proteins. The cleavage of PARP was detected with western blot analysis in a dose- and time-dependent manner. Simultaneously, GA treatment led to a decrease of the precursor forms of caspase−3, −8 and −9, as well as an increase of the active forms of caspase−3, −8 and −9, matching the pattern of PARP cleavage (Figure 2A). These data suggest that GA trigger DLBCL cell apoptosis likely *via* caspase activation.

It is well known that mitochondria are the regulating center of apoptosis. Release of cytochrome C and AIF from mitochondria to the cytoplasm is recognized as an indicator of the early stage of apoptosis^22^. As shown in Figure 2B, the integrity of mitochondrial membranes was decreased in both SU-DHL-4 and SU-DHL-2 cells after GA treatment. Moreover, after GA treatment, elevated levels of cytosolic cytochrome C and AIF, and reciprocally decreased levels of mitochondrial cytochrome C and AIF, were detected in a time-dependent manner in these two cell lines (Figure 2C).

To further understand the mechanism of GA-induced apoptosis, the effects of GA on the expression of other apoptosis-related proteins were measured. As shown in Figure 2D, GA decreased the level of anti-apoptotic proteins XIAP and Survivin in both SU-DHL-4 and SU-DHL-2 cells. The level of proapoptotic protein Bax increased in both cell lines, with less remarkable changes in the expression of Bcl-2. We also found that the level of anti-apoptotic protein myeloid cell leukaemia-1 (Mcl-1) was increased in the case of short-term or low-dose of GA treatment, but was still decreased with increasing doses and extension of time. We further observed that administration of pan-caspase inhibitor z-VAD-fmk prevented most GA-mediated decreases of XIAP but not Mcl-1 (Figure 2E). These results demonstrate that GA-induced caspase activation is required for the downregulation of anti-apoptotic protein XIAP.

### GA inhibits proteasome function in GCB- and ABC-DLBCL cells

**Figure 3:**
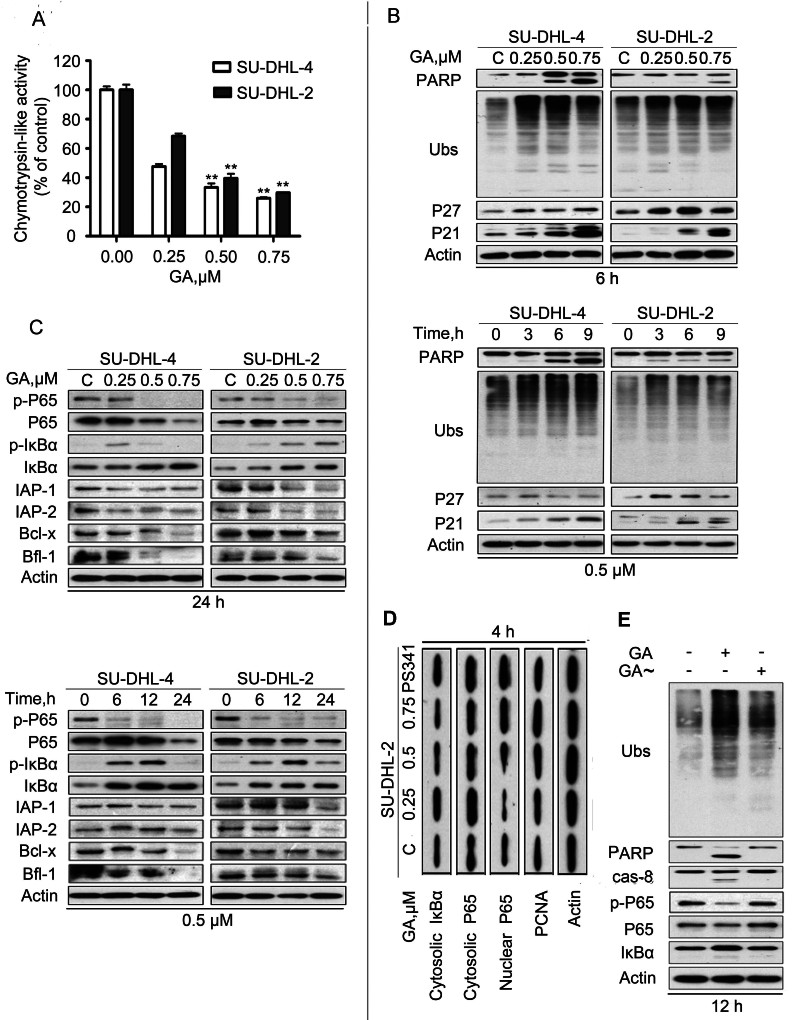
GA inhibits proteasome function in SU-DHL-4 and SU-DHL-2 cells. (A) GA inhibits proteasome peptidase activities in SU-DHL-4 and SU-DHL-2 cells. The cells were treated with GA at 37°C for 6 hours, followed by detecting CT-like activity with a Cell-Based Assay Reagent. Mean ± SD (n = 3). (B) GA accumulates proteasome substrate proteins in SU-DHL-4 and SU-DHL-2 cells. Cells were treated with GA at the indicated dose for the indicated time. The protein levels of PARP, ubiquitinated proteins (Ubs), p27 and p21 were detected with western blot analyses. Actin was used as a loading control. C: control. (C) GA up-regulates the expression of IκBα and decreases the protein levels of p65 subunit of NF-κB and its target proteins including IAP-1, IAP-2, Bcl-x and Bfl-1. SU-DHL-4 and SU-DHL-2 cells were treated with GA at the indicated dose for the indicated time. Western blot analyses were performed for detecting total and phosphorylated p65 and IκBα, as well as IAP-1, IAP-2, Bcl-x and Bfl-1. (D) GA and PS341 do not decrease the nuclear translocation of p65. SU-DHL-2 cells were treated with 0.25, 0.5, 0.75 μM GA and PS341 (50 nM) for 4 hours, cytoplasmic and nuclear proteins were extracted. Western blot analyses were performed for detection of the indicated proteins. Actin and PCNA were used as cytoplasm and nuclear protein loading controls, respectively. (E) GA-mediated proteasome inhibition is the major cause of either caspase activation or NF-κB downregulation. SU-DHL-2 cells were treated with GA (0.5 μM) for 12 hours and a C9-C10-disrupted GA (GA~) was used as a negative control. Western blot analyses were performed for detection of the indicated proteins. Actin was used as a loading control.

As reported in other cancer cells^19^,^44^, we found that GA dose- and time-dependently inhibited proteasome function in both GCB- and ABC-DLBCL cell lines. We first examined the proteasome peptidase activities in cultured GCB- and ABC-DLBCL cells. We found that GA dose-dependently inhibited the chymotrypsin-like activities in SU-DHL-4 and SU-DHL-2 cells (Figure 3A). Furthermore, GA induced accumulation of ubiquitinated proteins (Ubs) and proteasome substrate proteins p27 and p21 in SU-DHL-4 and SU-DHL-2 cells (Figure 3B). Mcl-1 can be degraded by the proteasome. Also corroborating a proteasome inhibition action by GA, Mcl-1 protein levels were discernibly increased in cells with low dose or short time GA treatment (Figure 2D, 2E). These results confirm that GA at a low concentration can significantly inhibit proteasome function in these DLBCL cells, associated with induction of cytotoxicity (Figure 1).

### GA downregulates the protein but not mRNA levels of some of the NF-κB target genes

We also found that GA treatment up-regulates the expression of IκBα which is an important proteasome substrate protein (Figure 3C). Further results showed that GA down-regulated the total and phosphorylation levels of the p65 subunit of NF-κB proteins in SU-DHL-4 and SU-DHL-2 cells in both a dose- and time-dependent manner (Figure 3C). To further determine the role of NF-κB inhibition in the proapoptotic activity of GA, we analyzed the effect of this compound on the protein and mRNA levels of some of the NF-κB target genes involved in cell survival, including IAP1, IAP-2, Bcl-x and Bfl-1. The results showed that GA down-regulated IAP1, IAP-2, Bcl-x and Bfl-1 protein expression to some extent during the long-term or high-dose GA treatment (Fig. 3C); however, real time PCR analyses failed to detect significant decreases in the mRNA level of these genes (supplementary data).

As NF-κB should enter the nucleus to exert its activities^23^, we then examined the translocation of NF-κB into the nucleus. As shown in Figure 3D, neither PS341 nor various doses of GA consistently decreased the nuclear presence of the NF-κB p65 subunit in SU-DHL-2 cells.

### GA-mediated proteasome inhibition is required for caspase activation

Next we investigated whether GA-mediated proteasome inhibition is responsible for caspase activation and NF-κB downregulation. We reported previously that a double bond between carbon 9 (C_9_) and carbon 10 (C_10_) in GA structure is the major chemical structure required for GA-induced proteasome inhibition^19^. In the current study, a C_9_-C_10_-disrupted GA (GA~) was used to compare with GA. As shown in Figure 3E, GA~ (0.5 μM) lost its ability to induce proteasome inhibition, caspase activation, apoptosis and NF-κB downregulation, compared with GA treatment in SU-DHL-2 cells. These results show that GA-mediated proteasome inhibition is responsible for GA-induced caspase activation.

### GA downregulates the protein levels of cell growth related signaling pathway

**Figure 4:**
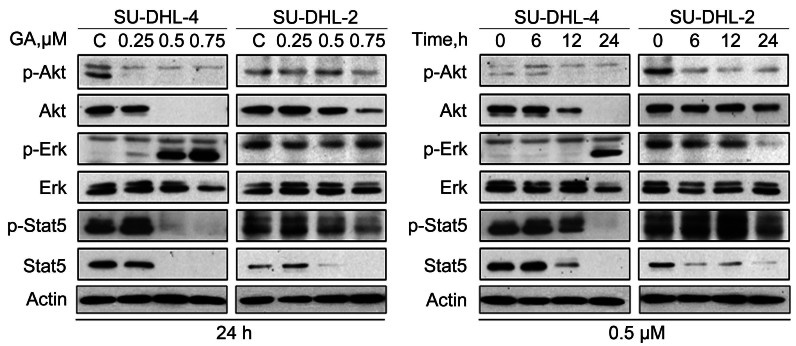
GA decreases the signaling protein levels of the cell growth related pathway, such as AKT, Erk1/2 and STAT5. SU-DHL-4 and SU-DHL-2 cells were treated with GA at the indicated dose for the indicated time. Cell lysates were analyzed using western blot. C: control.

As signal transduction pathways including the MAPK/ERK cascade, PI3K/Akt, and STATs are generally considered to promote tumor cell growth^24^,^25^,^26^, we also investigated the effect of GA on the protein expression of these signaling pathways in SU-DHL-4 and SU-DHL-2 cells. The phosphorylation of AKT, Erk1/2 and Stat5 were significantly decreased in a dose- and time-dependent manner with a less dramatic change in the total expression of Erk1/2 (Figure 4), indicating that GA suppress major cell growth signaling pathways, corroborating the data shown in Figure 1A and B in demonstrating a inhibitory effect of GA on DLBCL cell proliferation.

### GA restrains the growth of xenografted GCB- and ABC-DLBCL cells in nude mice

**Figure 5:**
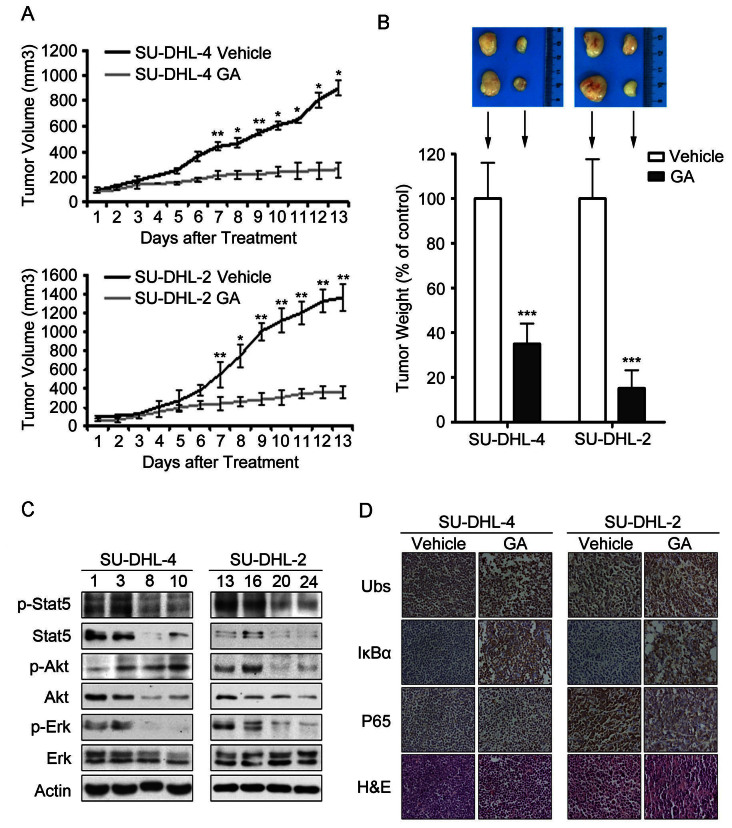
GA inhibits tumor growth in both GCB- and ABC-DLBCL xenografted mouse models. Nude BALB/c mice bearing SU-DHL-4 and SU-DHL-2 cells were randomized to vehicle- and GA (3 mg/kg/2d)-treatment group. Treatment was initiated when the average tumor size reached 50 mm^3^. (A) Tumor volume was recorded every day after treatment. Mean ± SD (n = 6). **P < 0.01, ***P < 0.001. (B) On day 13 after inoculation, the mice were sacrificed and the tumor tissues were weighed and imaged. ***P < 0.001 vs. the control group. (C) Cell growth related pathway in tumor tissues were detected with western blot analyses (SU-DHL-4 control group: 1, 3; GA-treated group: 8, 10; SU-DHL-2 control group: 13, 16; GA-treated group: 20, 24.) (D) Immunohistochemistry analyses were performed to examine ubiquitinated proteins, IκB-α, and P65 in the tumor tissues. All the immunostaining and western blot analyses were repeated in three mouse tumor tissues and the representative images are shown.

We next evaluated the *in vivo* effects of GA using a nude mouse xenograft model. In the *in vivo* model, SU-DHL-4 and SU-DHL-2 cells were inoculated subcutaneously in nude mice. Mice were then treated by *i.p* injection with vehicle or GA (3 mg/kg/2d) for 13 days. It was found that GA treatment significantly inhibited the growth of both GCB- and ABC-DLBCL xenografts; the weights of tumors were significantly reduced in GA-treated group compared to the vehicle-treated (Figs. 5A and B), while body weight remained relatively stable in each group (data not shown). Protein levels including Akt, Erk1/2, Stat5 (Figure 5C) and the p65 subunit of NF-κB proteins (Figure 5D) were significantly decreased in the GA-treated tumors, while proteasome target protein IκB-α and the ubiquitinated proteins were highly accumulated in GA-treated tumors *versus* the control (Figure 5D), indicating that GA inhibits proteasome function in both GCB- and ABC-DLBCL xenografts. Together, the results demonstrate that GA inhibits both xenografted GCB- and ABC-DLBCL cells *in vivo*.

## Discussion

DLBCL can be classified into three distinct subtypes (ABC-, GCB- and PMBCL- types) *via* gene expression profiling^2^,^3^,^4^. Improvements in DLBCL treatment, such as the introduction of R-CHOP therapy program^8^, have gradually appeared. Unfortunately, resistance to R-CHOP therapy and other current treatment regimens develops over time and is an emerging problem for DLBCL treatment^9^,^10^,^11^. Particularly, ABC-DLBCL, characterized by increased dependence on the NF-κB pathway, has poorer overall survival than the GCB-DLBCL counterpart^7^. Therefore, innovative therapeutic strategies for DLBCL patients, especially the ABC-DLBCL patients, are critically warranted.

GA, the major active component in the traditional Chinese medicine gamboge, has antitumor activities in a broad range of human cancer cells^13^,^14^. The results of the present study indicate that GA dramatically induces cytotoxicity in DLBCL cells, including both ABC- and GCB-DLBCL. In the cell culture experiments, GA dose- and time-dependently decreased cell viability, cell proliferation and induced cell death in both ABC- and GCB-DLBCL cell lines; in the *in vivo* experiment, both ABC- and GCB-DLBCL xenografted tumors were all sensitive to GA treatment. To our knowledge, this is the first report to show that GA is effective against DLBCL cells, including ABC-DLBCL cells.

Proteasome inhibitors such as carfilzomib have been reported to induce cell death in DLBCL cells^20^; however, the role of GA in DLBCL was not reported. Recently we have reported that GA is a tissue-specific proteasome inhibitor with low toxicity^19^. In the current study we discovered a pathway that GA-mediated proteasome inhibition and caspase activation is responsible for GA-induced cytotoxicity in DLBCL cells. Like in other cancer cells^19^,^44^, GA induced typical proteasome inhibition in both ABC- and GCB-DLBCL cells *in vitro* and *in vivo*. We have reported that proteasome inhibition-induced Bax accumulation plays an important role in proteasome inhibition-mediated caspase activation and cell apoptosis^27^. Here we found that GA induced Bax accumulation while anti-apoptotic proteins such as XIAP and Survivin were significantly decreased in a dose- and time-dependent manner. The imbalance between pro-apoptotic and anti-apoptotic factors led to the decrease of mitochondrial membrane integrity, thereby inducing the release of cytochrome C and AIF. The released apoptotic factors either directly or by forming a caspase-9 complex, induced caspase activation (Figure 2).

By proteasome inhibition, GA on one hand may induce caspase activation and then apoptosis; on the other hand, it may interfere with the degradation of IκBα and thereby prevents the activation of NF-κB. Normally, without stimulation, NF-κB bounds with IκBα, an important substrate protein in cytoplasm. With the extra- or intracellular stimulation, IκBα is phosphorylated and degraded by the ubiquitin-proteasome system, which frees out NF-κB in cytoplasm and allows its translocation into the nucleus to regulate cell survival and cell death^28^,^29^,^30^. To determine whether caspase activation is required for GA to downregulate anti-apoptotic proteins, cells were treated with GA (0.5 μM) for 12 hours in the absence or presence of z-VAD, then both XIAP (IAP family) and Mcl-1 (Bcl-2 family) proteins were detected by western blot. It was found that GA could decrease XIAP levels but increase Mcl-1 levels at the dose of 0.5 μM for 12 hours. XIAP decrease by GA was nearly completely recovered in the presence of a pan-caspase inhibitor z-VAD. Once the cells were treated with GA for more than 24 hours, Mcl-1 was also decreased, but the decrease could only be partially reversed by z-VAD (data not shown), consistent with a previous report^31^. These results suggest that the decrease of XIAP by GA depends primarily on the presence of caspase activation, inferring that transcriptional mechanisms mediated by pathways such as the NF-κB pathway may not play a significant role here.

To further clarify whether GA-mediated decreases of several other proteins depend suppression of the NF-κB pathway, we further detected the mRNA levels of several other target genes of the NF-κB pathway including IAP family members (IAP-1, IAP-2) and Bcl-2 family members (Bcl-x and Bfl-1). Surprisingly, we did not detect any obvious decreases in the mRNA expression after GA treatment (Supplementary Figure), suggesting that GA treatment induced downregulation of these proteins occurs as result of post-transcriptional mechanisms. Based on these results, the decrease of survivin at low dose of GA treatment or at short time point is probably not caused by a decrease in NF-κB activity as survivin is also a member of the IAP family. Moreover, we compared the effect of various doses of GA on NF-κB nuclear translocation with that of a classical proteasome inhibitor PS341. As shown in Figure 3D, low dose of GA (0.25 μM) did show the tendency to block the NF-kB nuclear translocation, but the other doses of GA as well as PS341 could not block the nuclear translocation of NF-kB. Taken together, our data suggest that inhibition of the NF-κB pathway does not appear to be a major mechanism for GA to induce apoptosis, contradictory to part of our original hypothesis.

Because of the critical role of the NF-κB signaling pathway, a previous report investigated the effects of GA on NF-κB-mediated cellular responses and NF-κB-regulated gene products in human leukemia cells. Treating the cells with GA enhanced apoptosis induced by tumor necrosis factor (TNF) and chemotherapeutic agents, and inhibited the expression of gene products involved in antiapoptosis (IAP-1 and IAP-2, Bcl-2, Bcl-x_L_, and TRAF1), cell proliferation (cyclin D1 and c-Myc), invasion (COX-2 and MMP-9), and angiogenesis (VEGF), all of which are known to be regulated by NF-κB^32^. However, in that report, they did not detect the mRNA expression after GA treatment alone. Here, our present study is the first time to report that GA did not downregulate mRNA expression of these NF-κB target genes in the cell lines tested here.

Our data show that GA accumulated Mcl-1 protein in the cell in the case of low dose of GA or short-time treatment, in agreement with what previously reported for other proteasome inhibitor treatment^33^. Mcl-1 protein degradation is mediated by proteasome- and/or caspase-dependent mechanisms. Both processes rapidly decrease its cellular level^34^,^35^. It has been reported that Mcl-1 strongly determines the effectiveness of bortezomib, a classical proteasome inhibitor. Mcl-1 accumulated in CD34 (+) AML cells upon bortezomib treatment and inhibition of Mcl-1 by shRNA significantly improved the sensitivity of CD34(+) AML cells to bortezomib. These results suggest that combining bortezomib with specific Mcl-1 inhibitors might potentially target the leukemic stem cells^36^. In our current study, one reason for Mcl-1 accumulation is likely its degradation inhibition mediated by GA-induced proteasome inhibition. Another possible reason is related to GA-mediated unfolded protein response (UPR). We have also previously reported that GA could induce UPR^19^. It was recently reported that Mcl-1 accumulation could be induced by the UPR, where the translation of activating transcription factor-4 (ATF4), an important effector of the UPR, was greatly enhanced by proteasome inhibition. ChIP analysis further revealed that bortezomib stimulated binding of ATF4 to a regulatory site (at position −332 to −324) at the promoter of the Mcl-1 gene. Knocking down ATF4 resulted in down-regulation of Mcl-1 in bortezomib-treated cells and significantly increased bortezomib-induced apoptosis. These studies identify the UPR and more specifically, its ATF4 branch as an important mechanism mediating up-regulation of Mcl-1 by proteasome inhibition^37^.

Notably, although Mcl-1 accumulation attenuates the pro-apoptotic effect of bortezomib, it is probably cannot do so to GA. Based on our results, GA efficiently induced cell death in these two cell lines even in the presence of Mcl-1 accumulation. This is possibly due to the direct effect of GA on Bcl-2 family proteins, in contrast to bortezomib which does not have such effect. It has been reported by others that suppression of antiapoptotic Bcl-2 family proteins may be a cytotoxic mechanism by which GA kills tumor cells. Using the antiapoptotic Bcl-2 family protein, Bfl-1, as a target for screening of a library of natural products, GA was identified as a competitive inhibitor that displaced BH3 peptides from Bfl-1. Analysis of competition for BH3 peptide binding revealed that GA inhibits all six human Bcl-2 family proteins to various extents, with Mcl-1 being most potently inhibited^38^. Hence, GA may be more potent and optimal than conventional proteasome inhibitor (eg., bortezomib) to kill cancer cells considering the important role of Mcl-1 in cell survival.

PI3K/AKT, Raf/Erk and Jak/STAT signal pathways are shown constitutive activation in many tumors, contributing to uncontrolled tumor cell growth^24^,^25^,^26^,^30^,^39^,^40^. Our results showed that GA induced decreases in the phosphorylation status of AKT, Erk1/2 and STAT5 (Figure 4), corroborating the impaired growth of GA-treated DLBCL cells (Figure 1A and 1B). The STAT5 enhancing survival of cancer cells involves the transcription of Mcl-1, survivin or XIAP^41^,^42^. Treatment with GA resulted in downregulation of Mcl-1, survivin and XIAP (Figure 2D). Even though GA may impact on multiple molecules, downregulation of these signaling proteins is at least one of the major factors to induce cell growth inhibition and apoptosis in DLBCL cells.

In summary, our findings collectively demonstrate that GA has a significant effect against the GCB and ABC subtypes of DLBCL cells *in vitro* and *in vivo*. Proteasome inhibition-induced caspase activation may chiefly contribute to GA-induced cytotoxicity in these cells. These findings suggest for the first time that GA may have clinical benefit for patients with DLBCL, particularly the patients with ABC subtypes DLBCL, which is of great importance in future exploration of clinical cancer therapy.

## Methods

### Chemicals

GA, diethyl dithiocarbamate (DDC), Annexin V, propidium iodide (PI) and rhodamine-123 were obtained from Sigma-Aldrich (St. Louis, MO). Mitochondria Isolation Kit was obtained from Thermo Scientific (TMO, USA).C_9_–C_10_ disrupted GA (GA~) was synthesized by our laboratory^19^. Antibodies (Abs) against Mcl-1 (S-19), ubiquitin (P4D1), caspase−3, −8, −9, apoptosis-inducing factor (AIF), P27, P21, Bcl-2 and Bax were from Santa Cruz Biotechnology (Santa Cruz, CA). Abs against poly (ADP)-ribose polymerase (PARP, clone 4C10-5) was from BD Biosciences. Abs against p65, phospho-p65 at Ser536, inhibitor of kappa B α (IκBα), phospho-IκBα at Ser32, phospho-Erk1/2 (T202/Y204), Erk1/2, phospho-Akt, Akt, IκB-α, cleaved caspase−3, −9, cytochrome C, Survivin, XIAP, IAP-1, IAP-2, Bcl-x and Bfl-1 were from Cell Signaling Technology (Beverly, MA, USA). Abs against phospho-Stat5A/B (Y694/Y699, clone 8-5-2) and Stat5 were from Upstate Technology; mouse monoclonal antibody against Actin, Cox-4 and PCAN from Sigma-Aldrich. Enhanced chemiluminescence reagents were purchased from Amersham Biosciences (Piscataway, NJ, USA).

### Cell culture

The DLBCL cell line SU-DHL-4 (GCB-DLBCL) and SU-DHL-2 (ABC-DLBCL) cells were purchased from ATCC and incubated in RPMI 1640 medium (Life Technologies) supplemented with 10% fetal calf serum (Hyclone), 1 unit/ml penicillin, and 1 μg/ml streptomycin. Cells were incubated at 37°C and in water vapor–saturated air with 5% CO_2_ at one atmospheric pressure.

### Preparation of cell fractions and western blot analysis

Whole cell lysates were prepared in RIPA buffer^21^ (1 × PBS, 1% NP-40, 0.5% sodium deoxycholate, 0.1% SDS) supplemented with 10 mM β-glycerophosphate, 1 mM sodium orthovanadate, 10 mM NaF, 1 mM phenylmethylsulfonyl fluoride (PMSF), and 1 × Roche Protease Inhibitor Cocktail (Roche, Indianapolis, IN). To detect the level of cytochrome C, AIF and NF-κB nucleus translocation, the cytosolic fraction was prepared with a digitonin extraction buffer (10 mM PIPES, 0.015% digitonin, 300 mM sucrose, 100 mM NaCl, 3 mM MgCl_2_, 5 mM EDTA, and 1 mM PMSF), and the nuclear protein was prepared with extraction buffer with inhibitors (20 mM Hepes, pH 7.9, 0.4 M NaCl, 1 mM EDTA with 1 mM DTT, 0.5 mM PMSF, 1 mM NaF and 1 mM Complete Protease Inhibitor Mix) as described previously^43^. To detect the level of mitochondrial cytochrome C and AIF, cells were extracted with Thermo Scientific Mitochondria Isolation Kit using the reagent-based method. The mitochondrial fractions were prepared in RIPA buffer supplemented with 10 mM NaF, 1 mM PMSF, and 1 × Roche Protease Inhibitor Cocktail. Western blotting was performed as we previously described^43^,^44^.

### Cell viability assay

MTS assay (CellTiter 96 Aqueous One Solution reagent, Promega) was used to measure cell viability^45^. Briefly, 2 × 10^5^/ml cells in 100 μl were treated with GA for 48 hours. Control cells received DMSO for a final concentration the same as the highest concentration of GA but less than 0.1%v/v. 4 hours before culture termination, 20 μl MTS was added to the wells. The absorbance density was read on a 96-well plate reader at wavelength 490 nm.

### Cell counting assay

SU-DHL-4 and SU-DHL-2 cells were seeded into 24-well plates (2 × 105/ml, 1 ml/well) and treated with GA in various concentrations for indicated duration, then 0.4% trypan blue (Sigma-Aldrich) was added to count the number of live and dead cells under a light microscope.

### Cell death assay

1.0% PI was added to the culture medium to monitor temporal changes in the incidence of cell death in the live culture condition. The PI-positive cells were imaged with an epi-fluorescence microscope equipped with a digital camera (Axio Obsever Z1, Zeiss, Germany)^45^. Cell apoptosis was determined by flow cytometry using Annexin V-fluoroisothiocyanate (FITC)/PI double staining^43^. SU-DHL-4 and SU-DHL-2 cells were collected, washed with binding buffer (Sigma-Aldrich, St. Louis, MO), and then incubated in working solution (100 μl binding buffer with 0.3 μl Annexin V-FITC and PI) for 15 minutes in dark.

### Measurement of mitochondrial membrane integrity

The mitochondrial membrane potential of GA-treated and untreated cells were assayed by using rhodamine-123 (Sigma-Aldrich, St. Louis, MO) staining. Cells were treated with various concentrations of GA for 24 hours and stained with 1 μM of rhodamine-123 for 1 hour at 37°C. Following the staining, the cells were washed and harvested for either flow cytometry analysis or imaging with an inverted fluorescence microscope.

### Chymotrypsin-like (CT-like) peptidase activity assay

About 4,000 SU-DHL-4 and SU-DHL-2 cells were treated with GA for 6 hours. The cells were then incubated with the Glo Cell-Based Assay Reagent (Promega Bioscience, Madison, WI) for 10 minutes^45^. The proteasomal CT-like activity was detected as the relative light unit (RLU) generated from the cleaved substrate. Luminescence generated from each reaction was detected with luminescence microplate reader (Varioskan Flash 3001, Thermo, USA).

### RNA isolation and real-time quantitative polymerase chain reaction (PCR)

Total RNA was extracted from 5 × 10^6^ cells by use of Trizol reagent (Invitrogen). After quantification by spectrophotometry, the first-strand cDNA was synthesized from 500 ng of total RNA with the use of the RNA PCR Kit (AMV) Ver.3.0 (TaKaRa, Dalian, China) and random primers. Then 50 ng of total cDNA was use for real-time PCR with the SYBR Premix Ex TaqIIKit (TaKaRa). The reaction used the ABI 7500 Real-Time PCR System. The relative gene expression was analyzed by the Comparative Ct method using 18 S ribosomal RNA as endogenous control, after confirming that the efficiencies of the target and the endogenous control amplifications were approximately equal. The specific primers for real-time PCR are as follows: IAP-1 forward, 5′-AAC ATG CCA AGT GGT TTC CAA-3′; IAP-1 reverse, 5′- TGA AGA ACT TTC TCC AGG TCC AA-3′; IAP-2 forward, 5′- AAG CCA GTT ACC CTC ATC TAC TTG-3′; IAP-2 reverse, 5′- GCT TCT ACT AAA GCC CAT TTC CA-3′; Bcl-x forward, 5′- CTG GCT CCC ATG ACC ATA CTG A-3′; Bcl-x reverse, 5′- GTG AGG CAG CTG AGG CCA TAA-3′; Bfl-1 forward, 5′- TCC GTA GAC ACT GCC AGA ACA C-3′; Bfl-1 reverse, 5′- CTC CGT TTT GCC TTA TCC ATT C-3′; 18 s forward, 5′-AAA CGG CTA CCA CAT CCA AG-3′; 18 s reverse, 5′-CCT CCA ATG GAT CCT CGT TA-3′.

### Nude mouse xenograft model

Nude Balb/c mice were bred at the animal facility of Guangzhou Medical College. The mice were housed in barrier facilities with a 12 hours light dark cycle, with food and water available ad libitum. 3 × 10^7^ of SU-DHL-4 and SU-DHL-2 cells were inoculated subcutaneously on the flanks of 5-week-old male nude mice. After 5–6 days of inoculation, mice were treated with either vehicle (10% DMSO, 30% cremophor and 60% NaCl) and GA (3 mg/kg/2 d) for totally 13 days. Tumors were measured and tumor volumes were calculated by the following formula: *a*^2^ × *b* × 0.4, where *a* is the smallest diameter and *b* is the diameter perpendicular to *a*. At day 13 after treatment, tumor xenografts were removed, weighed, stored and fixed. All experiments were performed in accordance with relevant guidelines and regulations. All animal studies were conducted with the approval of the Institutional Animal Care and Use Committee of Guangzhou Medical University.

### Statistical analysis

All experiments were performed at least thrice, and the results were expressed as mean ± SD where applicable. GraphPad Prism 4.0 software (GraphPad Software) was used for statistical analysis. Comparison of multiple groups was made with one-way ANOVA followed by Tukey's test or Newman-Kueuls test. *P* value of < 0.05 was considered statistically significant.

## Supplementary Material

Supplementary Information Supplementary information
