# Supplementary material for: Gambogic acid induces apoptosis in diffuse large B-cell lymphoma cells via inducing proteasome inhibition
Source: Sci Rep. 2015 Apr 8;5:9694. doi: 10.1038/srep09694 (PMC4894437; doi:10.1038/srep09694)
Supplement: Supplementary Information [file srep09694-s1.doc]

**Supplementary dataset**

**Gambogic acid induces apoptosis in diffuse large B-cell lymphoma cells *via* inducing proteasome inhibition**

**Running title:** Gambogic acid induces apoptosis in DLBCL cells

**Xianping Shi^1,4^, Xiaoying Lan^1,4^, Xin Chen^1^, Chong Zhao^1^, Xiaofen Li^1^, Shouting Liu^1^, Hongbiao Huang^1^, Ningning Liu^1, 2^, Dan Zang^1^, Yuning Liao^1^, Peiquan Zhang^1^, Xuejun Wang^1,3^ and Jinbao Liu^*,1^**


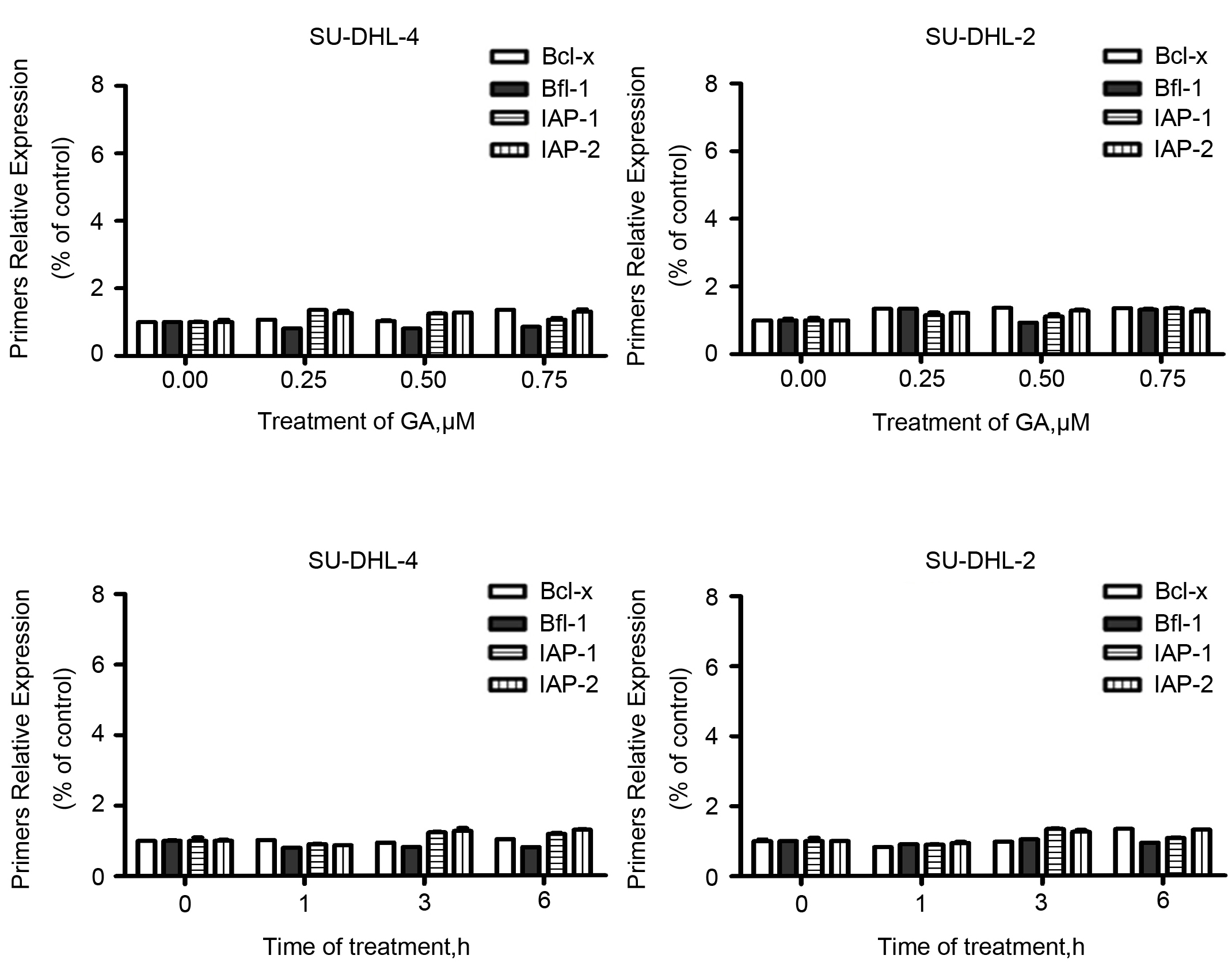


**Supplementary Figure**  Treatment with GA did not significantly suppress mRNA expression of NFκB target genes. SU-DHL-4 and SU-DHL-2 cells were exposed to 0.25, 0.5, 0.75 µM GA for 6 hours or 0.5 µM GA for 1, 3, 6 hours. The IAP-1, IAP-2, Bcl-x and Bfl-1 mRNA expression were measured by RT-qPCR and their expression levels relative to the control were presented. Mean±SD (n=3).
